# Right Ventricle in Arterial Hypertension: Did We Forget Something?

**DOI:** 10.3390/jcm11216257

**Published:** 2022-10-24

**Authors:** Marijana Tadic, Cesare Cuspidi

**Affiliations:** 1 Klinik für Innere Medizin II, Universitätsklinikum Ulm, Albert-Einstein Allee 23, 89081 Ulm, Germany; 2 Department of Medicine and Surgery, University of Milano-Bicocca, 20126 Milano, Italy

**Keywords:** right ventricle, arterial hypertension, speckle tracking, cardiac magnetic resonance

## Abstract

Right ventricular remodeling has been neglected in patients with arterial hypertension as all studies have concentrated on the left ventricle and left atrial-ventricular and ventricular-arterial coupling. The development of novel imaging techniques has revealed significant impairment in the RV structure, systolic and diastolic function, and, afterwards, RV longitudinal mechanics. However, these changes are subclinical and can be detected only after comprehensive imaging analysis. The latest findings confirm the importance of RV hypertrophy, systolic, and diastolic dysfunction in the prediction of cardiovascular adverse events in the hypertensive population, representing an important clinical implication of these parameters. In clinical practice, 2D echocardiography is widely used for the evaluation of RV remodeling. However, existing techniques are largely underused and limited to a few basic parameters (RV thickness and TAPSE), which are not nearly enough for a detailed assessment of RV remodeling. In addition, 3D echocardiography provides the possibility of accurate evaluation of RV volumes and ejection fraction, which are comparable with results obtained by cardiac magnetic resonance (CMR)—a gold standard for the evaluation of the RV. The use of 3D echocardiography is limited due to its low availability, the lack of adequate software necessary for the calculation of results, and the necessity for a higher level of expertise. CMR provides all information required for a detailed assessment of RV structural, functional, and mechanical remodeling, and it is considered the reference method for this type of evaluation. Furthermore, it is the only technique that may provide tissue characterization and evaluation of the interstitial space, which is essential for hypertensive heart disease. The aim of this review is to provide the current level of evidence regarding RV remodeling in patients with arterial hypertension evaluated with different imaging techniques and various parameters from each method.

## 1. Introduction

The right ventricle (RV) has long been considered unessential in patients with arterial hypertension. In patients with other conditions that are directly or indirectly involved with the RV, the importance of this cardiac chamber has already been well established, and assessment is provided in everyday clinical echocardiographic or CMR examinations. Nevertheless, studies published in the last decade have revealed the involvement of the RV in patients with arterial hypertension, including functional and mechanical changes, but also structural remodeling that has never before been related to arterial hypertension [1,2,3].

RV remodeling seems to follow changes on the left side, which means that subtle mechanical changes and diastolic dysfunction occur at the very beginning, while RV hypertrophy and subclinical impairment in systolic function come afterwards. These changes are important as they have been proven to be the independent predictors of cardiovascular and overall mortality in the general population as well as some specific groups of patients [4,5,6].

The imaging and assessment of different aspects of RV remodeling are challenging and have been long limited to 2D echocardiography, which enables only the basic parameters regarding RV structure and function. There are a couple of reasons for this. First, the anatomical position of the RV in the chest and the relationship with surrounding anatomical structures, as well as its specific crescent shape, do not facilitate the visualization and evaluation of all segments. The development of new techniques within 2D echocardiography, such as speckle tracking imaging, and the introduction of new imaging modalities, namely, 3D echocardiography and cardiac magnetic resonance (CMR), significantly increased our ability to assess the same parameters that are available for the LV. The huge scientific development in the imaging of the RV, achieved in the last decade, opened Pandora’s box and imposed the question of the clinical relevance of all parameters that we can measure nowadays. 

The current review article summarizes the current clinical and scientific approach in the assessment of RV structure, function, and mechanics in patients with arterial hypertension, which is an important but often neglected topic.

## 2. Hemodynamic Changes in Arterial Hypertension Related to RV Remodeling

Arterial hypertension is characterized by the overstimulation of the sympathetic system and the renin–angiotensin–aldosterone system (RAAS) as well as oxidative stress and endothelial dysfunction, all of which may induce impaired vascular relaxation [7,8,9]. RAAS is associated with fibroblast proliferation and an increase in interstitial and perivascular collagen of the LV, resulting in LV interstitial fibrosis, which represents a cornerstone of hypertension-induced LV remodeling. It is reasonable to hypothesize that similar changes induced by RAAS also occur on the right side, affecting the RV structure. The density of angiotensin II receptors is the same in the RV as in the LV of a healthy heart [10]; however, RAAS inhibition improved the RV myocardial performance index independently of BP reduction in patients with mild hypertension [11]. The MESA trial revealed that the use of RAAS inhibitors was related to changes in RV morphology, independent of LV effects [12].

Ventricular interaction is another important reason that explains RV remodeling in arterial hypertension, as hemodynamic changes are transferred from the LV to the RV through the interventricular septum. Buckberg et al. called the septum “*the lion of the right ventricular function*” and it is already known that the septum contributes to approximately 30% of RV systolic function [13]. Therefore, it is logical to assume that increased pressure and elevated wall stiffness in patients with arterial hypertension may transfer from the LV to the RV.

Some reports have revealed increased pulmonary pressure in patients with arterial hypertension. This can be explained by several mechanisms: (i) the transmission of increased systemic pressure in hypertensive patients combined with the elevation of pulmonary microvascular resistance; (ii) the retrograde transmission of impaired LV relaxation, diastolic dysfunction, and hemodynamic changes. This increase does not usually lead to the development of pulmonary hypertension that would be diagnosed by echocardiography but to an increase in pulmonary pressure compared with normotensive controls.

## 3. Structural RV Changes

The RV differs from the LV by several anatomical features: pronounced trabeculation, a muscular strand that connects the interventricular septum to the anterior papillary muscle called a moderator band, and a lack of fibrous continuity between its inflow and outflow valves. It has helical fibers distributed into epicardial and endocardial layers, which perform torsion and provide more effective RV systolic function. However, the RV does not have a middle layer and, therefore, relies more on the RV longitudinal shortening than the LV. Even though the interventricular septum is considered a part of the LV, it also includes longitudinal fibers that belong to the RV. It is clear that fibers from different layers in the LV and RV strongly interact and provide effective biventricular function.

A recently published study has investigated macroscopic and microscopic differences in the RV structure between hypertensive patients and controls [14]. The authors reported the significantly higher thickness of the RV anterior and posterior walls in hypertensive patients than in controls, but there was no difference in dimensions between these groups. Microscopic examination revealed only the hypertrophy of cardiomyocytes in RV hypertrophic walls, but there was no interstitial fibrosis or any other impairment of the spatial arrangement of muscle fibers [14]. 

Echocardiographic studies showed significantly higher RV wall thickness in patients with arterial hypertension than in controls [15], which was also confirmed by CMR [16]. Some investigations went a step further and studied the relationship between RV mass and inflammation parameters such as C-reactive protein (CRP), interleukin -6 (IL-6), and fibrinogen [17]. The influence of RAAS on the RV structure in hypertensive patients was assessed indirectly through the usage of angiotensin-converting enzyme inhibitors (ACEIs) or angiotensin II receptor blockers (ARBs) [12]. The authors found that the use of these medications was associated with lower RV mass in African Americans after adjustment for multiple covariates, including LV mass, whereas their usage was related to larger RV end-diastolic volume after adjustment for LV volume only among Caucasians [12]. Data derived from the same study (the Multi-Ethnic Study of Atherosclerosis—MESA) revealed that total arterial compliance and systemic vascular resistance were inversely associated with CMR-derived RV volumes and RV mass in patients without clinically evident cardiovascular disease [3]. The authors explained their results by the fact that the usage of a single vascular parameter, such as total arterial compliance and systemic vascular resistance, underestimates the LV workload due to the complex LV–vascular coupling. Total arterial compliance, calculated by the ratio of stroke volume and arterial pulse pressure, represents a surrogate measure of pulsatile arterial load. It is in an inverse relationship with vascular stiffness and lower total arterial compliance, which means more vascular stiffness, and has been associated with lower LV mass and higher LV wall-to-cavity volume ratio in a MESA study [18]. Similar findings were reported for the RV in this study. However, this trial included participants from the general population and not only hypertensive patients, which may have influenced the final results.

On the other hand, 2D and 3D echocardiographic studies showed a direct correlation between systolic blood pressure and RV wall thickness in hypertensive patients as well as a relationship between systolic blood pressure and RV dilatation, confirming the negative impact of arterial hypertension and even prehypertension on RV remodeling [19,20,21]. These changes in RV thickness and 3D RV volume were negatively correlated with peak oxygen consumption—the surrogate of functional capacity in the hypertensive population. Our recently published study showed that RV hypertrophy was an independent predictor of adverse events (atrial fibrillation, myocardial infarction, myocardial revascularization, heart failure, stroke, or cardiovascular death) in the hypertensive population during a follow-up of 9 years [5]. These findings emphasize the importance of RV structural remodeling in hypertensive patients. Table 1 summarizes all necessary information about the technical advantages and disadvantages of 2D and 3D echocardiography, as well as CMR, in the evaluation of RV and RA remodeling in patients with arterial hypertension. 

CMR studies revealed significant changes in interstitial myocardial tissue not only when using conventional techniques such as late gadolinium enhancement [22,23]; even more pronounced interstitial fibrosis was detected by novel methods that provide tissue characterization—T1 mapping and calculated extracellular volume [24,25]. These findings refer to LV remodeling in hypertensive heart disease, but there is no reason why similar changes would not be detected on the right side. The main issue is that the current programs used for T1 mapping and the calculation of extracellular volume are dedicated to the assessment of the LV.

## 4. Functional Remodeling

RV functional changes in arterial hypertension are usually subclinical but detectible with sophisticated imaging methods. This remodeling considers systolic and diastolic dysfunction. Conventional parameters of RV systolic function (TAPSE, FAC, and s’) normally do not reveal any impairment in systolic function in hypertensive patients. On the other hand, pulsed and tissue Doppler parameters of RV diastolic function often reveal diastolic dysfunction in hypertensive patients. The most relevant point is which imaging technique (echocardiography vs. CMR) as well as which method within a technique (2D and 3D echocardiography, M-mode, Doppler, or speckle tracking) should be used for the evaluation of RV function. The sensitivity, specificity, and reproducibility of the diverse techniques are different, and it is not difficult to understand the variations in results between studies.

## 5. Systolic Function

**Figure 1:**
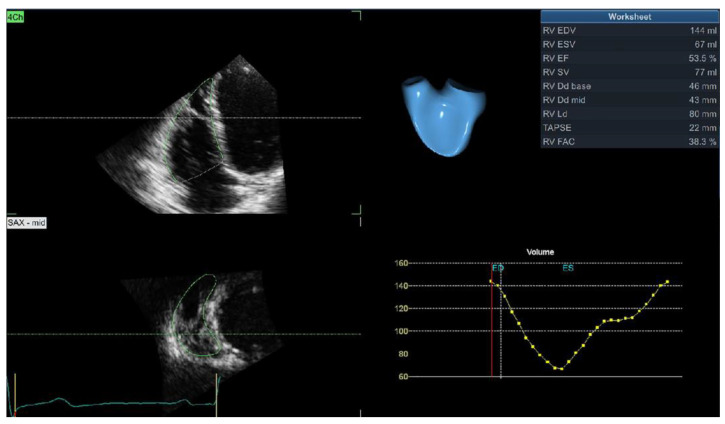
3D echocardiographic evaluation of right ventricular volume and ejection fraction.

Most of the published data showed no difference in conventional 2D echocardiographic parameters (TAPSE, s’, and FAC) between hypertensive and normotensive subjects [19,26,27]. However, new imaging modalities have revealed the deterioration of RV systolic function in hypertensive patients. Our study showed significantly reduced 3D RVEF in hypertensive patients [19]. We also reported that LV geometry has a significant impact on 3D RV volumes and RVEF and found that hypertensive patients with dilated LVH and combined concentric-dilated LVH had significantly lower 3D RVEF than hypertensive patients with normal LV geometry, concentric remodeling, and concentric or eccentric LVH [28]. Impaired RV systolic function is a predictor of adverse events in the hypertensive population, but its predictive importance vanishes after an adjustment for other clinical and echocardiographic parameters [5]. Figure 1 shows the 3D echocardiographic assessment of RV volumes and the ejection fraction.

## 6. Diastolic Function

The evaluation of RV diastolic function is not widely used in clinical practice as its clinical implications have been insufficiently investigated, and the guidelines are not as clear as for LV diastolic dysfunction. Parameters used in the evaluation of RV diastolic function are obtained by pulsed and tissue Doppler. Results from different echocardiographic evaluations are conflicting. Some authors found significantly impaired tricuspid E/A and E/e’ ratios in hypertensive patients [19,20,28,29,30]. Pedrinelli et al. reported a gradual reduction in peak early diastolic tricuspid value with an increase in BP [26] but did not find any difference in tricuspid E/A between hypertensive and controls [23,24,26,27]. Our study showed that RV diastolic function gradually deteriorated from patients with optimal BP to high-normal BP to hypertensive patients [21]. Findings showed an association between 24 h systolic BP and tricuspid E/e’, independent of clinical and echocardiographic parameters [20,21]. RV diastolic dysfunction was associated with adverse cardiovascular events but was not an independent predictor when the model included other echocardiographic and clinical parameters [5].

**Figure 2:**
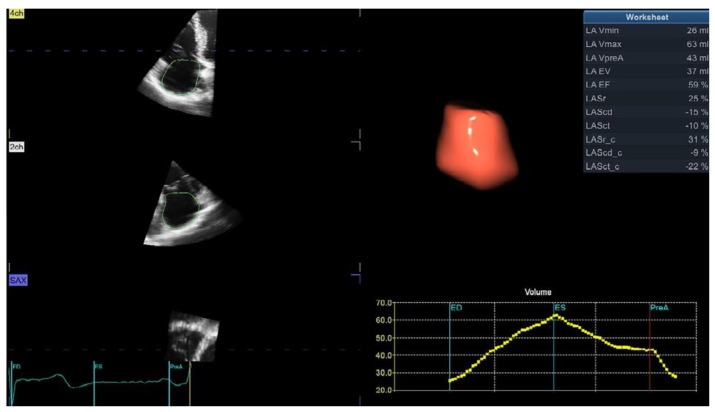
Three-dimensional echocardiographic volume- and strain-derived right atrial phasic analysis (software is dedicated for left atrial analysis and, therefore, all acronyms are devoted to the left atrium (LA) and should be replaced with the right atrium (RA) as the same software is used off-label for the RA). ED—end-diastole, ES—end-systole, LA EV—left atrial emptying volume, LA EF—left atrial emptying fraction, LASr—left atrial longitudinal strain during reservoir phase, LAScd—left atrial longitudinal strain during conduit phase, LASct—left atrial longitudinal strain during contraction phase, LASr_c—left atrial circumferential strain during reservoir phase, LAScd_c—left atrial circumferential strain during conduit phase, LASc_ct—left atrial circumferential strain during contraction phase, LA Vmax—maximum left atrial volume, LA Vmin—minimum left atrial volume, LA VpreA—left atrial volume before atrial contraction, pre-A—before atrial contraction.

The right atrium (RA) is enlarged in hypertensive patients, and our study showed that the phasic function is significantly influenced by systemic BP [21]. RA phasic function gradually deteriorated from patients with optimal BP, across patients with high-normal BP, to those with arterial hypertension [21]. A study that investigated the influence of different LV geometric patterns reported that RA conduit and reservoir function gradually decreased and RA pump function gradually increased from hypertensive patients with normal LV geometry to those with LV dilatation and concentric LVH [31]. RA dilatation and dysfunction significantly contribute to RV diastolic dysfunction in hypertensive patients and may explain changes in RV filling pressures assessed by tricuspid E/A and E/e’. Nevertheless, RA enlargement was not proven to be an independent predictor of adverse events in hypertensive patients [5]. New 3D echocardiographic software provides a rapid and simultaneous evaluation of RA volumes and volume-derived phasic function (Figure 2).

## 7. RV Mechanics in Arterial Hypertension

Currently used echocardiographic techniques do not provide the same comprehensive evaluation of RV mechanics that is available for the LV. RV mechanics evaluation is normally limited to longitudinal strain, whereas circumferential and radial strain rates are available only in CMR-derived analyses. Moreover, 3D echocardiographic evaluation of RV mechanics, providing data on longitudinal, circumferential, radial, and area strain rates, is available from a limited number of vendors (Toshiba), and the data are scarce and limited to patients with pulmonary hypertension [32]. Longitudinal strain may be evaluated for the whole visible part of the RV (interventricular septum and free wall) in the apical 4-chamber view or only for the RV free wall, which some authors consider as the only representative for RV mechanics as it does not include the septum, a part of the LV. The assessment of RV mechanics is not part of clinical examinations yet, only clinical research. 

**Figure 3:**
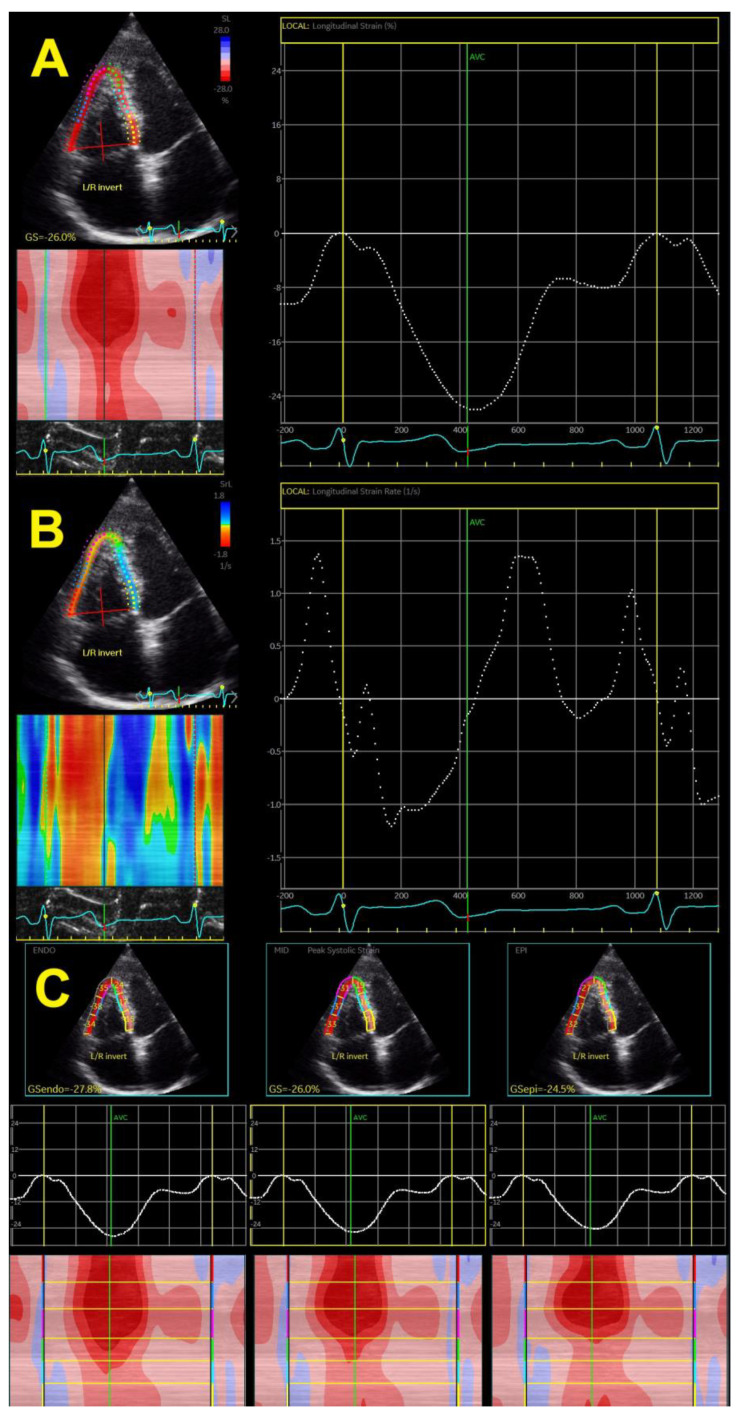
Two-dimensional global right ventricular strain (**A**), right ventricular strain rate (**B**), and layer-specific strain—endocardial, mid-myocardial, and epicardial (**C**).

The speckle tracking-derived RV longitudinal strain has been the only method used in the last decade (Figure 3A), whereas the tissue-Doppler-derived strain has not been used for evaluation for a long time due to many limitations. This technique also provides a calculation of strain rates (systolic, early, and late diastolic strain rates) (Figure 3B), which is the speckle tracking equivalent to parameters obtained by tissue Doppler, giving data on systole and early and late diastole. Novel echocardiographic methods enable the assessment of epicardial and endocardial RV longitudinal strain rates (Figure 3C), which has also been used in the hypertensive population.

Early findings revealed that the free wall RV longitudinal strain was gradually reduced with systolic BP [27]. The same was reported for the early diastolic strain rate but not for late diastolic and systolic strain rates. Our study group showed that RV GLS and, particularly, free wall RV longitudinal strain were deteriorated in patients with different forms of hypertension, such as white-coat hypertension, masked hypertension, and nocturnal hypertension, but also in patients with high-normal BP [21,33,34,35,36]. Patients with unfavorable circadian BP patterns (non-dipping and reverse dipping) also have significantly lower values of RV longitudinal strain in comparison with dipping and extreme dipping BP patterns [33]. RV GLS also gradually reduced from hypertensive patients with normal geometry and concentric remodeling, across those with eccentric and dilated LVH, to those with concentric and concentric-dilated LVH [28].

Patients with comorbidities such as obesity, diabetes, and metabolic syndrome had worse RV GLS than patients with isolated hypertension [36,37,38,39], which shows that comorbidities have both additive and cumulative negative effects on RV mechanics. Studies also revealed that the RV endocardial layer was more impacted by arterial hypertension than the epicardial layer in hypertensive patients; this was also found in hypertensive patients with diabetes and obesity [34] and patients with white-coat or masked hypertension [33,34]. Systolic BP correlates well with layer-specific RV longitudinal strain, independent of other clinical and echocardiographic parameters [33,34,38].

Data regarding CMR-derived RV GLS are scarce, and one study reported significantly lower RV GLS in diabetic patients with and without arterial hypertension compared with controls, but there was no difference between diabetic patients with and without diabetes [40].

RA longitudinal strain is also significantly decreased in hypertensive patients compared with controls [20,21]. It is also worse in non-dippers than in dippers and in patients with concentric and dilated-concentric LVH than in patients with normal LV geometry or concentric remodeling [31]. It is clear that RA mechanical changes follow RV remodeling; this is of great importance for understanding pathophysiological findings in right atrioventricular coupling in hypertensive patients. The clinical implication of this finding might be the association between RA enlargement and dysfunction with the occurrence and recurrence of atrial fibrillation [41], which is especially frequent among hypertensive patients. Timely detection of RA enlargement and dysfunction might predict patients who are at higher risk of developing this arrhythmia and enable their regular monitoring and diagnosis of atrial fibrillation; this would significantly reduce the risk of stroke or even prevent atrial fibrillation by an aggressive antihypertensive treatment that could result in reverse remodeling and a decrease in RA volume.

**Figure 4:**
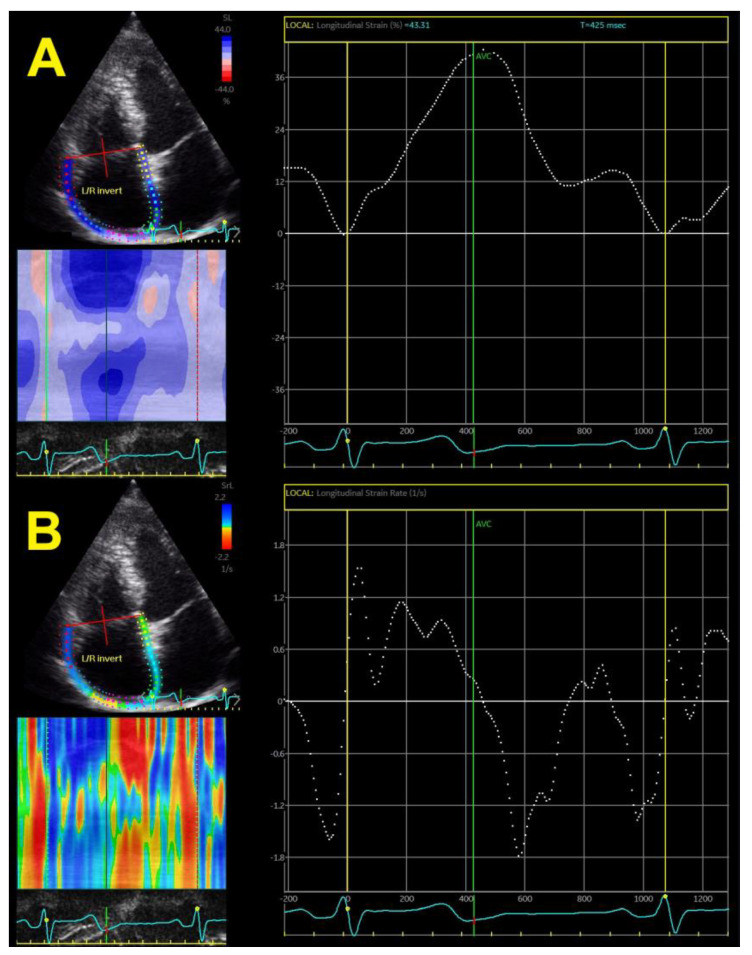
Two-dimensional right atrial strain (**A**) and strain rates (**B**).

RA phasic function, determined by different segments of the longitudinal strain curve, showed impairment that correlated with systolic BP. Strain-derived RA phasic function is faster, easier, and more reliable and reproducible than volume-derived RA phasic function and, therefore, might be easily used in clinical practice and not only for research purposes (Figure 4A). RA strain rates provide additional information about the RA phasic function (Figure 4B).

## 8. Clinical Usage of Different Imaging Modalities

The evaluation of RV remodeling is challenging, even with new imaging modalities, but it is significantly improved and provides more comprehensive information than only a decade ago. Conventional echocardiography should be used for the evaluation of RV structure (thickness), systolic parameters (TAPSE, s’, and FAC), diastolic dysfunction (E/A, E/e’), and RA evaluation (volumetric-derived phasic function). Our recent longitudinal study showed that RV hypertrophy, RV systolic dysfunction, RV diastolic dysfunction, and RA dilatation were correlated with adverse cardiovascular events in the hypertensive population during a 9-year follow-up [5]. However, after adjustment for demographic and clinical parameters, only RV hypertrophy remained an independent predictor of these events [5]. The MESA study showed that CMR-calculated RV hypertrophy was associated with the risk of heart failure or mortality in a multi-ethnic population free of clinical cardiovascular disease at baseline but with a high percentage of patients with arterial hypertension [4]. A recent study showed that RA enlargement was associated with higher LVMI and LA dilatation in well-controlled hypertensive patients, but the influence of RA on outcomes was not investigated [42].

Speckle tracking echocardiography has opened a new era in RV and RA imaging as it is a convenient, reasonably fast, and reproducible method that provides not only information about the current status of right heart mechanics but has an important predictive value in a large number of cardiovascular conditions [43,44]. Therefore, it is reasonable to expect the same clinical implication value in patients with arterial hypertension. Speckle tracking applied for the RV provides RV GLS, strain rates, and layer-specific strain, whereas the same technique applied to the RA provides GLS and strain-derived phasic function. Three-dimensional echocardiography enables the rapid assessment of RV and RA volumes and systolic function, comparable with CMR, which also facilitates the detailed evaluation of RV and RA remodeling in everyday clinical practice, even though the availability of 3D echocardiography and appropriate software represents a significant limitation. CMR provides the most comprehensive information regarding RV structure, including tissue characterization, RV volumes, and systolic function (RVEF), as well as RV mechanics (2D longitudinal, circumferential, and radial strain rates). However, CMR is not expected to be used in the assessment of hypertensive heart disease in the near future as it is still reserved for more complex cases with differential diagnoses that cannot be completely excluded by echocardiography. Table 1 summarizes all the technical aspects and possibilities that current echocardiography and CMR provide in the evaluation of hypertensive patients.

## 9. Conclusions

New imaging modalities have revealed a significant involvement of the RV and RA in patients with arterial hypertension. Even though these changes are subtle and subclinical, they are related to adverse outcomes in hypertensive patients, and this is why they should be diagnosed in a timely fashion and regularly followed. RV longitudinal strain should be included in the clinical reports of patients with arterial hypertension as it provides comprehensive information about RV systolic function and mechanics and has important predictive value. Larger studies that include hypertensive patients with longer follow-up periods are warranted to demonstrate the clinical utility of RV (and RA) GLS in this population of patients.

## Figures and Tables

**Table 1:** Advantages and disadvantages of different imaging modalities in the evaluation of right ventricular and right atrial remodeling in patients with arterial hypertension.

	2DE	3DE	CMR
**Technical aspects**			
Availability	++++	++	+
Cost	+	++	++++
Level of competence	++	+++	++++
Typical scan duration (min)	10	<5*	35-40
Dependence on acoustic windows	++++	++++	/
**RV structure, volume, and systolic function**			
Estimation of RV wall thickness	++	+++	++++
Determination of RV diameters	++	+++	++++
RV volume quantification	/	+++	++++
Calculation of RVEF calculation	/	++++	++++
Evaluation of RV diastolic function	++++	/	+
**RV mechanics**			
2D GLS strain	++++	/	++
3D strain			
Longitudinal	/	++	++++
Circumferential	/	++	++++
Radial	/	++	/
Area	/	++	/
**RA volumes and function**	++++	++++	++++
**RA longitudinal strain**	++++	/	+++

+—low, ++—moderate, +++—high, ++++—very high, *—additional time necessary after 2DE, 2DE—two-dimensional echocardiography, 3DE—three-dimensional echocardiography, EF—ejection fraction, GLS—global longitudinal strain, RA—right atrium, RV—right ventricle, RVEF—right ventricular ejection fraction.

## Data Availability

Not applicable.

## References

[1] Zhang X., Wu C.E., Ye P., Sheng L., Luo L. (2019). Right ventricle may be involved in regional diastolic dysfunction earliest in primary hypertension patients. J. Cell Biochem..

[2] Liu S., Liao Y., Zhu Z., Wang S., Li Y., Liang D., Xie Y., Zhang Z. (2022). Association between cumulative blood pressure in early adulthood and right ventricular structure and function in middle age: The CARDIA study. Clin. Cardiol..

[3] Al-Naamani N., Chirinos J.A., Zamani P., Ruthazer R., Paulus J.K., Roberts K.E., Barr R.G., Lima J.A., Bluemke D.A., Kronmal R. (2016). Association of systemic arterial properties with right ventricular morphology: The Multi-Ethnic Study of Atherosclerosis (MESA)-Right Ventricle Study. J. Am. Heart Assoc..

[4] Kawut S.M., Barr R.G., Lima J.A., Praestgaard A., Johnson W.C., Chahal H., Ogunyankin K.O., Bristow M.R., Kizer J.R., Tandri H. (2012). Right ventricular structure is associated with the risk of heart failure and cardiovascular death: The Multi-Ethnic Study of Atherosclerosis (MESA)—Right ventricle study. Circulation.

[5] Tadic M., Cuspidi C., Celic V., Petrovic O., Pencic B., Mancia G., Grassi G., Ivanovic B. (2020). The prognostic importance of right ventricular remodeling and the circadian blood pressure pattern on the long-term cardiovascular outcome. J. Hypertens..

[6] Dietz M.F., Prihadi E.A., van der Bijl P., Goedemans L., Mertens B.J.A., Gursoy E., van Genderen O.S., Ajmone Marsan N., Delgado V., Bax J.J. (2019). Prognostic implications of right ventricular remodeling and function in patients with significant secondary tricuspid regurgitation. Circulation.

[7] Jia G., Aroor A.R., Hill M.A., Sowers J.R. (2018). Role of renin-angiotensin-aldosterone system activation in promoting cardiovascular fibrosis and stiffness. Hypertension.

[8] Levick S.P., Murray D.B., Janicki J.S., Brower G.L. (2010). Sympathetic nervous system modulation of inflammation and remodeling in the hypertensive heart. Hypertension.

[9] Rababa’h A.M., Guillory A.N., Mustafa R., Hijjawi T. (2018). Oxidative stress and cardiac remodeling: An updated edge. Curr. Cardiol. Rev..

[10] Urata H., Healy B., Stewart R.W., Bumpus F.M., Husain A. (1989). Angiotensin II receptors in normal and failing human hearts. J. Clin. Endocrinol. Metab..

[11] Pechlivanidis G., Mantziari L., Giannakoulas G., Dimitroula H., Styliadis H., Karvounis H., Styliadis I.H., Parharidis G. (2011). Effects of renin–angiotensin system inhibition on right ventricular function in patients with mild essential hypertension. J. Renin-Angiotensin-Aldosterone Syst..

[12] Ventetuolo C.E., Lima J.A.C., Barr R.G., Bristow M.R., Bagiella E., Chahal H., Kizer J.R., Lederer D.J., Bluemke D.A., Kawut S.M. (2012). The renin-angiotensin system and right ventricular structure and function: The MESA-right ventricle study. Pulm. Circ..

[13] Buckberg G.D., RESTORE Group (2006). The ventricular septum: The lion of right ventricular function, and its impact on right ventricular restoration. Eur. J. Cardiothorac. Surg..

[14] Kosiński A., Piwko G.M., Kamiński R., Nowicka E., Kaczyńska A., Zajączkowski M., Czerwiec K., Gleinert-Rożek M., Karnecki K., Gos T. (2022). Arterial hypertension and remodelling of the right ventricle. Folia Morphol..

[15] Cuspidi C., Sala C., Muiesan M.L., De Luca N., Schillaci G., Working Group on Heart, Hypertension of the Italian Society of Hypertension (2013). Right ventricular hypertrophy in systemic hypertension: An updated review of clinical studies. J. Hypertens..

[16] Todiere G., Neglia D., Ghione S., Fommei E., Capozza P., Guarini G., Dell’omo G., Aquaro G.D., Marzilli M., Lombardi M. (2011). Right ventricular remodelling in systemic hypertension: A cardiac MRI study. Heart.

[17] Harhay M.O., Tracy R.P., Bagiella E., Barr R.G., Pinder D., Hundley W.G., Bluemke D.A., Kronmal R.A., Lima J.A., Kawut S.M. (2013). Relationship of CRP, IL-6, and fibrinogen with right ventricular structure and function: The MESA-Right Ventricle Study. Int. J. Cardiol..

[18] Zamani P., Bluemke D.A., Jacobs DRJr Duprez D.A., Kronmal R., Lilly S.M., Ferrari V.A., Townsend R.R., Lima J.A., Budoff M., Segers P. (2015). Resistive and pulsatile arterial load as predictors of left ventricular mass and geometry: The Multi-Ethnic Study of Atherosclerosis. Hypertension.

[19] Tadic M., Cuspidi C., Pencic B., Jozika L., Celic V. (2015). Relationship between right ventricular remodeling and heart rate variability in arterial hypertension. J. Hypertens..

[20] Tadic M., Cuspidi C., Suzic-Lazic J., Andric A., Stojcevski B., Ivanovic B., Hot S., Scepanovic R., Celic V. (2014). Is there a relationship between right-ventricular and right atrial mechanics and functional capacity in hypertensive patients?. J. Hypertens..

[21] Tadic M., Cuspidi C., Pencic B., Sljivic A., Ivanovic B., Neskovic A., Scepanovic R., Celic V. (2014). High-normal blood pressure impacts the right heart mechanics: A three-dimensional echocardiography and two-dimensional speckle tracking imaging study. Blood Press. Monit..

[22] Puntmann V.O., Jahnke C., Gebker R., Schnackenburg B., Fox K.F., Fleck E., Paetsch I. (2010). Usefulness of magnetic resonance imaging to distinguish hypertensive and hypertrophic cardiomyopathy. Am. J. Cardiol..

[23] Rudolph A., Abdel-Aty H., Bohl S., Boyé P., Zagrosek A., Dietz R., Schulz-Menger J. (2009). Noninvasive detection of fibrosis applying contrast-enhanced cardiac magnetic resonance in different forms of left ventricular hypertrophy relation to remodeling. J. Am. Coll. Cardiol..

[24] Kuruvilla S., Janardhanan R., Antkowiak P., Keeley E.C., Adenaw N., Brooks J., Epstein F.H., Kramer C.M., Salerno M. (2015). Increased extracellular volume and altered mechanics are associated with LVH in hypertensive heart disease, not hypertension alone. JACC Cardiovasc. Imaging.

[25] Wu L.M., An D.L., Yao Q.Y., Ou Y.Z., Lu Q., Jiang M., Xu J.R. (2017). Hypertrophic cardiomyopathy and left hypertrophy in hypertensive heart disease with mildly reduced or preserved ejection fraction: Insight from altered mechanics and native T1 mapping. Clin. Radiol..

[26] Pedrinelli R., Canale M.L., Giannini C., Talini E., Penno G., Dell’Omo G., Di Bello V. (2010). Right ventricular dysfunction in early systemic hypertension: A tissue Doppler imaging study in patients with high-normal and mildly increased arterial blood pressure. J. Hypertens..

[27] Pedrinelli R., Canale M.L., Giannini C., Talini E., Dell’Omo G., Di Bello V. (2010). Abnormal right ventricular mechanics in early systemic hypertension: A two-dimensional strain imaging study. Eur. J. Echocardiogr..

[28] Tadic M., Cuspidi C., Vukomanovic V., Kocijancic V., Celic V. (2016). Right ventricular remodeling and updated left ventricular geometry classification: Is there any relationship?. Blood Press..

[29] Maresca A.M., Mongiardi C., Corso R., Robustelli Test L., Lippi A., Montalbetti L., Campiotti L., Moretti S., Tandurella N., Agostinis M. (2020). Right ventricular remodelling in mild hypertensive patients: Role of left ventricular morpho-functional parameters. J. Hum. Hypertens..

[30] Cicala S., Galderisi M., Caso P., Petrocelli A., D’Errico A., de Divitiis O., Calabrò R. (2002). Right ventricular diastolic dysfunction in arterial systemic hypertension: Analysis by pulsed tissue Doppler. Eur. J. Echocardiogr..

[31] Tadic M., Cuspidi C., Kocijancic V., Celic V., Vukomanovic V. (2016). Does left ventricular geometric patterns impact right atrial phasic function? Findings from the hypertensive population. Echocardiography.

[32] Smith B.C., Dobson G., Dawson D., Charalampopoulos A., Grapsa J., Nihoyannopoulos P. (2014). Three-dimensional speckle tracking of the right ventricle: Toward optimal quantification of right ventricular dysfunction in pulmonary hypertension. J. Am. Coll. Cardiol..

[33] Tadic M., Cuspidi C., Ivanovic B., Vukomanovic V., Djelic M., Celic V., Kocijancic V. (2016). The impact of white-coat hypertension on cardiac mechanics. J. Clin. Hypertens..

[34] Tadic M., Cuspidi C., Vukomanovic V., Celic V., Pavlovic T., Kocijancic V. (2016). The influence of masked hypertension on the right ventricle: Is everything really masked?. J. Am. Soc. Hypertens..

[35] Tadic M., Cuspidi C., Celic V., Pencic-Popovic B., Mancia G. (2018). Nocturnal hypertension and right heart remodeling. J. Hypertens..

[36] Tadic M., Cuspidi C., Sljivic A., Pencic B., Mancia G., Bombelli M., Grassi G., Galderisi M., Kocijancic V., Celic V. (2020). Do reverse dippers have the highest risk of right ventricular remodeling?. Hypertens. Res..

[37] Tadic M., Cuspidi C., Vukomanovic V., Kocijancic V., Celic V., Stanisavljevic D. (2016). The association between obesity, blood pressure variability, and right ventricular function and mechanics in hypertensive patients. J. Am. Soc. Echocardiogr..

[38] Tadic M., Cuspidi C., Vukomanovic V., Ilic S., Celic V., Obert P., Kocijancic V. (2016). The influence of type 2 diabetes and arterial hypertension on right ventricular layer-specific mechanics. Acta Diabetol..

[39] Tadic M., Cuspidi C., Sljivic A., Andric A., Ivanovic B., Scepanovic R., Ilic I., Jozika L., Marjanovic T., Celic V. (2014). Effects of the metabolic syndrome on right heart mechanics and function. Can. J. Cardiol..

[40] Shao G., Cao Y., Cui Y., Han X., Liu J., Li Y., Li N., Liu T., Yu J., Shi H. (2020). Early detection of left atrial and bi-ventricular myocardial strain abnormalities by MRI feature tracking in normotensive or hypertensive T2DM patients with preserved LV function. BMC Cardiovasc. Disord..

[41] Xie E., Yu R., Ambale-Venkatesh B., Bakhshi H., Heckbert S.R., Soliman E.Z., Bluemke D.A., Kawut S.M., Wu C.O., Nazarian S. (2020). Association of right atrial structure with incident atrial fibrillation: A longitudinal cohort cardiovascular magnetic resonance study from the Multi-Ethnic Study of Atherosclerosis (MESA). J. Cardiovasc. Magn. Reson..

[42] Cicco S., Calvanese C., Susca N., Inglese G., Nardiello E., Ciampi S., Tedesco P.A., Cirulli A., Panettieri I., Vacca A. (2022). Right atrium enlargement is related to increased heart damage and mortality in well-controlled hypertension. Nutr. Metab. Cardiovasc. Dis..

[43] Tadic M., Nita N., Schneider L., Kersten J., Buckert D., Gonska B., Scharnbeck D., Reichart C., Belyavskiy E., Cuspidi C. (2021). The Predictive Value of Right Ventricular Longitudinal Strain in Pulmonary Hypertension, Heart Failure, and Valvular Diseases. Front. Cardiovasc. Med..

[44] Tadic M., Kersten J., Nita N., Schneider L., Buckert D., Gonska B., Scharnbeck D., Dahme T., Imhof A., Belyavskiy E. (2021). The Prognostic Importance of Right Ventricular Longitudinal Strain in Patients with Cardiomyopathies, Connective Tissue Diseases, Coronary Artery Disease, and Congenital Heart Diseases. Diagnostics.

